# Comparative effects of acupuncture and metformin on insulin sensitivity in women with polycystic ovary syndrome: a systematic review and meta-analysis

**DOI:** 10.3389/fendo.2025.1553684

**Published:** 2025-06-18

**Authors:** Yun Wang, Hongchu Bao, Jianxiang Cong, Qinglan Qu

**Affiliations:** Department of Reproductive Medicine, Yantai Yuhuangding Hospital Affiliated to Qingdao University, Yantai, Shandong, China

**Keywords:** acupuncture, polycystic ovary syndrome, metformin, insulin resistance, systematic review, meta-analysis

## Abstract

**Objective:**

To systematically evaluate the clinical efficacy of acupuncture and metformin on insulin resistance (IR) in women with Polycystic Ovary Syndrome (PCOS).

**Methods:**

A computer search was conducted up to September 30, 2024, in databases including PubMed, EMBASE, Cochrane Library, China Biology Medicine disc (CBM), China National Knowledge Infrastructure (CNKI), Wanfang, and VIP, to collect randomized controlled trials (RCTs) on acupuncture treatment for PCOS. The effect of acupuncture and metformin on insulin sensitivity in PCOS patients was assessed. Two researchers independently screened the literature, extracted data, and evaluated the quality of included studies using the bias assessment tool recommended in the Cochrane Handbook for Systematic Reviews of Interventions 5.1.0. Meta-analysis and network analysis were conducted using Stata 17.Our primary outcome measure was the homeostasis model assessment of insulin resistance(HOMA-IR).The secondary outcomes were fasting blood glucose (FBG), fasting insulin (FINS), body mass index (BMI), and waist-to-hip ratio (WHR).

**Results:**

A total of 11 RCTs involving 1,248 patients were included. The meta-analysis results showed that the reduction in HOMA-IR [SMD = 0.17, 95% CI (-0.18, 0.52)] and FINS [SMD = 0.17, 95% CI (-0.38, 0.71)] values in the acupuncture group were smaller than those in the metformin group. However, reductions in BMI [SMD = -0.15, 95% CI (-0.88, 0.58)], WHR [SMD = -0.15, 95% CI (-0.88, 0.58)], and FPG [SMD = -0.19, 95% CI (-0.40, 0.02)] were greater in the acupuncture group than in the metformin group, but the differences were not statistically significant. Subgroup analysis with placebo interventions added to each group showed that metformin plus sham acupuncture was more effective than acupuncture plus placebo in reducing HOMA-IR values [SMD = 0.49, 95% CI (0.02, 0.96)] with a statistically significant difference; acupuncture plus placebo treatment had a greater advantage in reducing FPG [SMD = -0.38, 95% CI (-0.57, -0.19)] with a statistically significant difference. The network meta-analysis results indicated that only Electroacupuncture (SMD = -0.27, 95% CI [-1.37, 0.83]) and Abdominal acupuncture (SMD = -0.13, 95% CI [-1.19, 0.94]) demonstrated significant effects on reducing HOMA-IR values in women with polycystic ovary syndrome (PCOS) compared to Metformin. However, the differences between groups were not statistically significant. Furthermore, based on SUCRA values, the advantages of various acupuncture modalities for interventions targeting HOMA-IR outcomes were ranked. Electroacupuncture was identified as the most effective intervention for lowering HOMA-IR values in women with PCOS-related insulin resistance (SUCRA value: 67.4%), followed by Abdominal acupuncture (SUCRA value: 58%).

**Conclusion:**

Metformin is more effective in improving HOMA-IR in patients with PCOS, while acupuncture has a greater advantage in reducing FPG. Among the different acupuncture modalities, Electroacupuncture emerged as the optimal intervention for reducing HOMA-IR values in insulin-resistant women with PCOS. Due to the limitations of existing studies, the conclusions of this study need to be confirmed by high-quality, large-sample, multicenter RCTs.

**Systematic review registration:**

https://www.crd.york.ac.uk/prospero/, identifier CRD42023381672.

## 1 Introduction

Polycystic ovary syndrome (PCOS) is a common reproductive endocrine disorder in women of reproductive age, the pathogenesis of which is not fully understood, with a population prevalence of 6%-10% (1, 2), and a heterogeneous clinical presentation that includes ovulation disorders, hyperandrogenemia, and polycystic changes in the ovaries, as well as metabolic dysfunction and psychological disorders (3, 4). The problem of untimely diagnosis and unsatisfactory treatment exists globally (5). Currently, the Rotterdam 2004 diagnostic criteria are still the most used. At least two of the following symptoms are required: oligomenorrhea/amenorrhea, excessive secretion of biochemical/clinical androgen, or polycystic ovary morphology (PCOM) (6). PCOS not only impacts the reproductive health of women of childbearing age but is also acknowledged as a metabolic disorder associated with significant long-term health risks. It has been linked to an elevated likelihood of developing insulin resistance (IR), impaired glucose tolerance (IGT), type 2 diabetes (T2D), dyslipidemia, and cardiovascular diseases (7). Notably, insulin resistance occurs in up to 95% of obese women with PCOS and up to 75% of lean women with PCOS (8). Insulin resistance and compensated hyperinsulinemia stimulate the production of excess testosterone by follicular membrane cells in the ovaries, leading to the clinical manifestations of hyperandrogenemia including acne, hirsutism, and alopecia, as well as an increased risk for type 2 diabetes mellitus in women with PCOS (9).

Metformin, an oral hypoglycemic agent that enhances glucose uptake by skeletal muscle and adipocytes by increasing insulin sensitivity, has been used as a first-line therapeutic agent for insulin resistance in patients with PCOS. However, the use of metformin may increase gastrointestinal side effects, and long-term metformin treatment may lead to lactic acidosis with poor patient compliance (3, 10), so other non-pharmacologic treatment modalities still need to be considered as alternative therapies.

Acupuncture is an important part of traditional Chinese medicine (TCM), and acupuncture is becoming increasingly popular as an alternative and complementary therapy in the world (11). Numerous clinical and animal experiments have shown (12–14) that PCOS patients benefit from receiving acupuncture treatment, which can improve ovulation disorders, insulin resistance and obesity, hyperandrogenemia, and mood disorders. Accumulated evidence from prior meta-analyses has corroborated the clinical benefits of acupuncture and metformin in addressing disorders of glucose and lipid metabolism as well as insulin resistance, with demonstrated improvements across key biomarkers including homeostasis model assessment (HOMA) index, fasting blood glucose (FBG), fasting insulin (FINS), body mass index (BMI), waist-to-hip ratio (WHR), and triglycerides (TG) (15–17). Nevertheless, there is still substantial heterogeneity in the magnitude of the reported effect. Therefore, the present study was conducted to systematically evaluate and meta-analyze the effects of acupuncture and metformin on insulin sensitivity in patients with PCOS to provide evidence-based evidence to support clinical treatment.

## 2 Methods

The analysis was performed in strict accordance with the PRISMA Statement (18). In addition, the evaluation is registered with PROSPERO under the registration number CRD42023381672. Available from: https://www.crd.york.ac.uk/PROSPERO/view/CRD42023381672.

### 2.1 Literature search

We conducted literature searches in seven Chinese and English databases: four English databases (PubMed, Embase, Cochrane Library, and Web of Science) and three Chinese databases (Wanfang, China Science and Technology Journal Database [VIP], and China Knowledge Network [CNKI]). From the time of library construction until September 30, 2024. No language restriction was imposed. The search keywords: “polycystic ovary syndrome”, “acupuncture”, “metformin”, etc., were performed using a combination of free words and subject terms. For detailed search strategies, please refer to **Supplementary Data Sheet 1**.

Literature screened from various databases was managed using EndNote (version 20) reference management software. The following characteristics of the included studies were summarized in a Microsoft Excel spreadsheet: year of publication, authors, population, intervention type, outcome measure, and control group.

### 2.2 Inclusion and exclusion criteria

#### 2.2.1 Inclusion criteria

(1) Study Design: Randomized controlled trial.

(2) Participants: This study includes patients diagnosed with polycystic ovary syndrome (PCOS). The inclusion criteria are based on the Rotterdam criteria established by the European Society of Human Reproduction and Embryology and the American Society for Reproductive Medicine (6), or the expert consensus on the diagnosis and treatment of PCOS issued by the Endocrinology Group of the Obstetrics and Gynecology Branch of the Chinese Medical Association (19). All participants who meet the eligibility criteria will be included, regardless of race or nationality.

(3) Intervention Type: The experimental group will receive acupuncture-based interventions, including acupuncture, needling, body acupuncture, electroacupuncture, moxibustion, abdominal acupuncture, acupuncture point embedding, and transcutaneous electrical acupoint stimulation, or a combination of acupuncture and metformin placebo treatment.

(4) Control Group Type: The control group will receive metformin treatment, or metformin combined with sham acupuncture treatment.

(5) Outcomes: At least one clinical outcome related to PCOS-associated insulin resistance (PCOS-IR) will be assessed. The primary outcome is the Homeostasis Model Assessment of Insulin Resistance (HOMA-IR). Secondary outcomes include fasting blood glucose (FBG), fasting insulin (FINS), body mass index (BMI), and waist-to-hip ratio (WHR). Safety outcomes are any adverse events.

Insulin resistance (IR) will be evaluated using the HOMA-IR index, calculated as: (FBG × FINS)/22.5, with a threshold value ≥ 2.14 (20).

(6) Publication Form: Journal article, thesis.

(7) Data Requirements: The study must include original data for the relevant outcome measures or data that can be extracted from numerical values and tables.

#### 2.2.2 Exclusion criteria

(1)Participants who are receiving treatments with medications other than metformin, such as antidiabetic drugs, statins, or other hormonal therapies.(2)Participants who are concurrently using traditional Chinese herbal medicine during the treatment.(3)Randomized controlled trials with incomplete abstract information or unclear outcome measures.(4)Animal studies.(5)Research published in the form of conference abstracts, theses, study protocols, or books.

### 2.3 Data extraction

Two researchers independently screened the articles identified through the search process. After an initial review of the titles and abstracts, full-text articles were further assessed according to the inclusion and exclusion criteria to determine the final set of studies for inclusion. Any uncertainties or disagreements were resolved through discussion or by consulting a third researcher.

The extracted data included:

1. Basic information of the study: title, authors, study design, publication date;
2. Key study details: participant characteristics, diagnostic criteria, sample size, and intervention methods;
3. Study outcomes: primary and secondary outcome measures, adverse effects, and any reported adverse events.

### 2.4 Assessment of quality and risk of bias

Two reviewers independently assessed the risk of bias in the included studies using the tool outlined in the Cochrane Handbook. The evaluation focused on several key domains: random sequence generation, allocation concealment, blinding of participants and personnel, blinding of outcome assessment, completeness of outcome data, selective reporting, and other potential biases. The risk of bias for each domain was categorized as low, high, or unclear. In cases of disagreement between the reviewers, consensus was reached through discussion with the corresponding author.

### 2.5 Data analysis

Data analysis was conducted using StataMP17 software to perform traditional meta-analysis, with the overall aim of evaluating the effects of acupuncture and metformin on insulin sensitivity in patients with polycystic ovary syndrome (PCOS). Heterogeneity was assessed using the Q-test and I² statistic. A value of I² ≤ 50% or P ≥ 0.1 was considered indicative of low heterogeneity, while I² > 50% or P < 0.1 suggested high heterogeneity. For studies with high heterogeneity, a random-effects model was applied, while a fixed-effects model was used for studies with low heterogeneity. In the presence of significant heterogeneity, subgroup analyses were performed based on intervention type. Sensitivity analyses were conducted for studies with high heterogeneity, where studies with the greatest influence on heterogeneity were sequentially removed from the model. The pooled effects on efficacy and safety before and after removal of these studies were compared, and statistical significance was assessed. Bayesian network meta-analysis was conducted using Stata 17.0. Continuous variables were expressed as mean differences (MD) with 95% confidence intervals (CIs). When the units of continuous variables were inconsistent, standardized mean differences (SMD) were employed to account for these discrepancies. In constructing the network evidence plot, the size of each node reflected the sample size for the respective intervention, while the strength of the connections between interventions indicated the number of randomized controlled trials (RCTs) comparing the two interventions. In the case of an open-loop network structure, a consistency model was applied. For closed-loop structures, inconsistency tests were performed to evaluate the consistency of outcome measures. When P > 0.05, indicating a high degree of consistency between direct and indirect evidence, the consistency model was retained. If significant heterogeneity was present, subgroup analysis and meta-regression were used to explore potential sources of variation. The surface under the cumulative ranking curve (SUCRA) was used to rank the effectiveness of non-pharmacological interventions across various outcome measures, with values ranging from 0 to 100; higher SUCRA values correspond to higher efficacy rankings. Additionally, for closed-loop structures, loop inconsistency tests were conducted to assess the consistency of each outcome measure. If the 95% CI of the loop inconsistency factor included 0, this indicated satisfactory consistency between direct and indirect evidence.

## 3 Results

### 3.1 Search results

**Figure 1:**
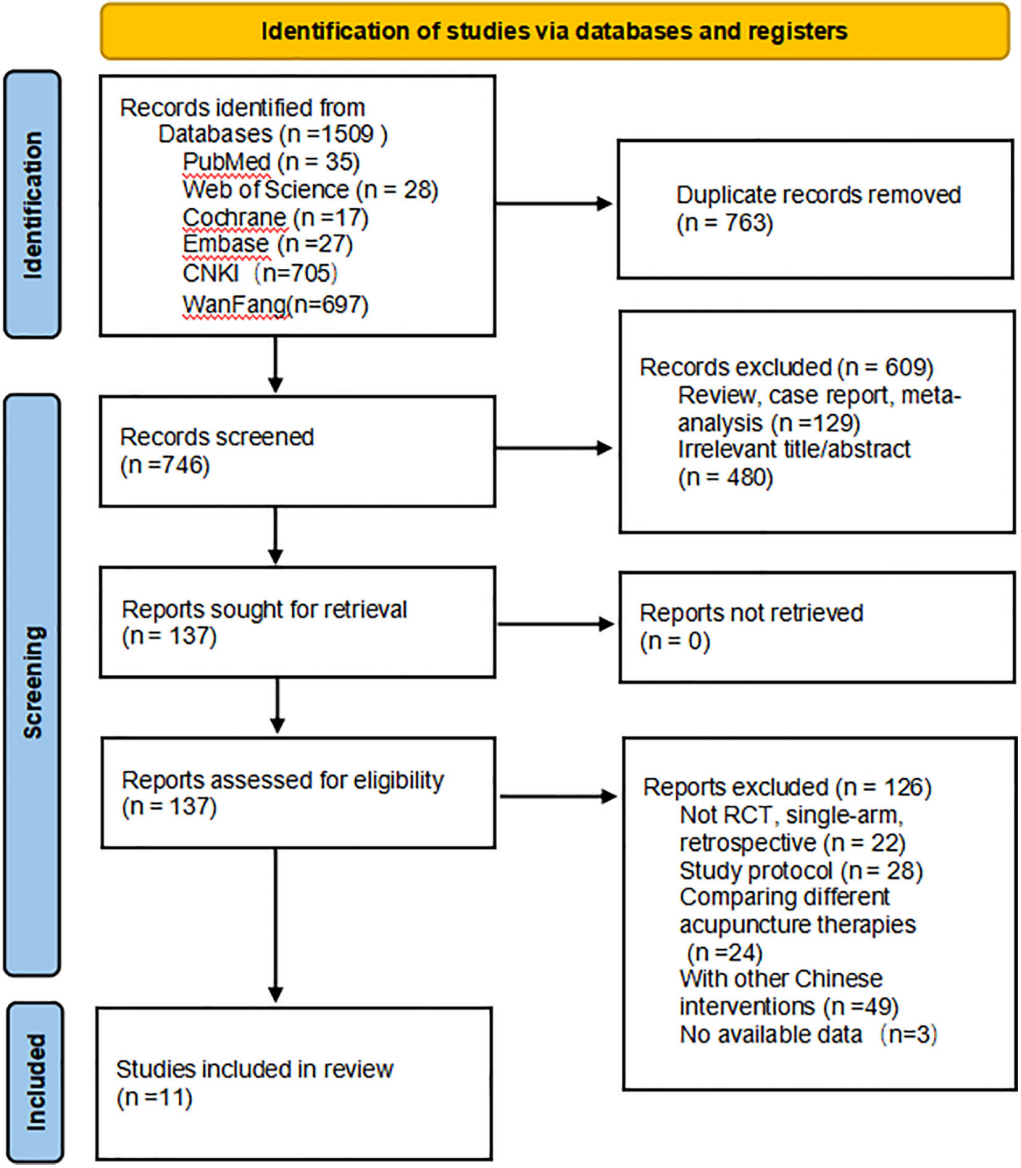
Literature screening process and results.

From the establishment of the database until September 2024, a literature search was conducted across seven databases. The initial search identified 1,509 articles. Using EndNote, 763 duplicate studies were excluded. After screening the titles and abstracts, 609 studies were further excluded. The full texts of the remaining 137 studies were assessed, and 126 studies were excluded for failing to meet the inclusion criteria. Additionally, three articles were excluded due to the unavailability of data, as we did not receive a response from the corresponding authors. (This is illustrated in the figure). Ultimately, 11 studies, involving 1,248 patients, were included in this review. The study selection process is detailed in **Figure 1**.

### 3.2 Characteristics of included studies

**Table 1** summarizes the characteristics of the 11 randomized controlled trials (RCTs) included in the review. Of these, three studies were indexed in English-language databases, while eight were indexed in Chinese-language databases. All participants in these studies were diagnosed with polycystic ovary syndrome (PCOS). Eight studies compared acupuncture (including abdominal acupuncture, acupoint embedding, electroacupuncture, etc.) with metformin (21–28), while three studies compared acupuncture combined with metformin placebo with metformin combined with sham acupuncture (30–32). The fundamental characteristics of these studies are summarized in **Table 1**. See appendix for details.

**Table 1:** Characteristics of included studies.

studyID	Country	Sample size (TG/CG)	Treatment	Control	Acupuncture points	Duration of intervention (months)	Age (average ±SD)	Diagnostic criteria	Outcomes
Wen, 2022 (29)	China	97/96	True acupuncture + Placebo	Metformin+sham acupuncture	Set1: Zhongji (CV3), Zhongwan (CV12), Guilai (bilateral) (ST29), Liangqiu (bilateral) (ST34), Yinshi (bilateral) (ST33), Sanyinjiao (bilateral) (SP6), Zusanli (bilateral) (ST36), Hegu (bilateral) (LI4); Set2: Daju (bilateral) (ST27), Qihai (CV6), Xiawan (CV10), Extra-meridian point (bilateral), Xuehai (bilateral) (SP10), Taichong (bilateral) (LR3), Neiguan (bilateral) (PC6), Sanyinjiao (bilateral) (SP6)	4	27.68±4.34 27.66±4.45	Rotterdam criteria	HOMA-IR, FBG, FINS, BMI, WHR, adverse events
Cao, 2023 (30)	China	62/65	True acupuncture + Placebo	Metformin+sham acupuncture	Set1: Zhongji (CV3), Zhongwan (CV12), Guilai (bilateral) (ST29), Liangqiu (bilateral) (ST34), Yinshi (bilateral) (ST33), Sanyinjiao (bilateral) (SP6), Zusanli (bilateral) (ST36), Hegu (bilateral) (LI4); Set2: Daju (bilateral) (ST27), Qihai (CV6), Xiawan (CV10), Extra-meridian point (bilateral), Xuehai (bilateral) (SP10), Taichong (bilateral) (LR3), Neiguan (bilateral) (PC6), Sanyinjiao (bilateral) (SP6).	4	28.40±4.42 28.76±4.08	Rotterdam criteria	HOMA-IR, FBG, FINS, BMI, WHR
Zheng, 2013 (21)	China	43/43	Abdominal acupuncture	Metformin	Guanyuan (CV4), Qihai (CV6), Xiawan (CV10), Zhongwan (CV12), Liangmen (ST21), Tianshu (ST25), Shuidao (ST28).	6	26.50±3.00 24.90±4.90	Rotterdam criteria	HOMA-IR, FBG, FINS, BMI, WHR, adverse events
Yu, 2020 (22)	China	31/30	Electroacupuncture	Metformin	Group 1 Main Acupoints: Yinlingquan (SP9), Zhongwan (CV12), Sanyinjiao (SP6), Zigong (EX-CA1), Zusanli (ST36), Taixi (KI3), Guanyuan (CV4), Tianshu (ST25), Daimai (GB26), Taibai (SP3), Qihai (CV6), Fenglong (ST40).Group 2 Main Acupoints: Diji (SP8), Sanjiaoshu (BL22), Yingu (KI10), Pishu (BL20), Ganshu (BL18), Yishu (EX-B3), Shenshu (BL23), Gongsun (SP4), Ciliao (BL32).	3	30.00±6.00 31.00±6.00	Rotterdam criteria	HOMA-IR, adverse events
He, 2020 (26)	China	62/60	Acupoint thread-embedding	Metformin	Group 1 Main Acupoints: Ganshu (BL18), Zhongji (CV3), Geshu (BL17), Zusanli (ST36), Sanyinjiao (SP6), Daimai (GB26), Guanyuan (CV4).Group 2 Main Acupoints: Shenshu (BL23), Pishu (BL20), Tianshu (ST25), Shuifen (CV9), Yinlingquan (SP9), Fenglong (ST40), Zigong (EX-CA1).	3	25.00±7.00 25.00±6.00	Chinese diagnosis and treatment guide for polycystic ovary syndrome	HOMA-IR, FBG, FINS
Lai, 2012 (24)	China	60/60	Abdominal acupuncture	Metformin	Zhongwan (CV12), Xiawan (CV10), Qihai (CV6), Guanyuan (CV4), Tianshu (ST25), Shuidao (ST28).	4	26.72±2.65 26.46±2.72	Rotterdam criteria	HOMA-IR, FBG, FINS, BMI, WHR
Li, 2014 (31)	China	49/51	True acupuncture + Placebo	Metformin+sham acupuncture	Zhongji ( CV3), Guanyuan (CV4), Zusanli (ST36), Sanyinjiao (SP6), Fuliu (KI7), and Qihai (CV6), Zigong (EX-CA1).	6	26.20±2.10 25.20±1.80	Rotterdam criteria	HOMA-IR, FBG, FINS, BMI, WHR, adverse events
Li, 2016 (23)	China	14/14	Abdominal acupuncture	Metformin	Tianshu (ST25), Zhongwan (CV12), Guanyuan (CV4), Qihai (CV6), Daheng (SP15), Huaroumen (ST24), Shuidao (ST28), Fuai (SP16), Fujie (SP14), Guilai (ST29).	6	Not available	Not specified	BMI, WHR
Liu, 2013 (25)	China	30/30	Acupuncture + embedding therapy	Metformin	Zhongwan (CV12), Xiawan (CV10), bilateral Tianshu (ST25), bilateral Daheng (SP15), bilateral Daimai (GB26), Qihai (CV6), Guanyuan (CV4), bilateral Shuidao (ST28), and bilateral Guilai (ST29).	3	Not available	Rotterdam criteria	BMI, WHR
Wang, 2009 (27)	China	30/30	Acupuncture + embedding therapy	Metformin	Bilateral Liangmen (ST21), Bilateral Tianshu (ST25), Bilateral Daimai (GB26), Bilateral Guilai (ST29), Bilateral Xuehai (SP10), Bilateral Sanyinjiao (SP6).	3	Not available	Rotterdam criteria	BMI
Yao, 2018 (28)	China	48/48	Acupuncture	Metformin	Danzhong (CV17), bilateral Ganshu (BL18), bilateral Tianshu (ST25), bilateral Zigong (EX-CA1), bilateral Zusanli (ST36), bilateral Qimen (LR14), Zhongwan (CV12), Guanyuan (CV4), bilateral Sanyinjiao (SP6), and bilateral Taichong (LR3).	6	27.80±4.80 28.20±4.50	Rotterdam criteria	HOMA-IR, BMI, WHR, adverse events

TG, treatment group; CG, control group.

### 3.3 Risk of bias assessment

**Figure 2:**
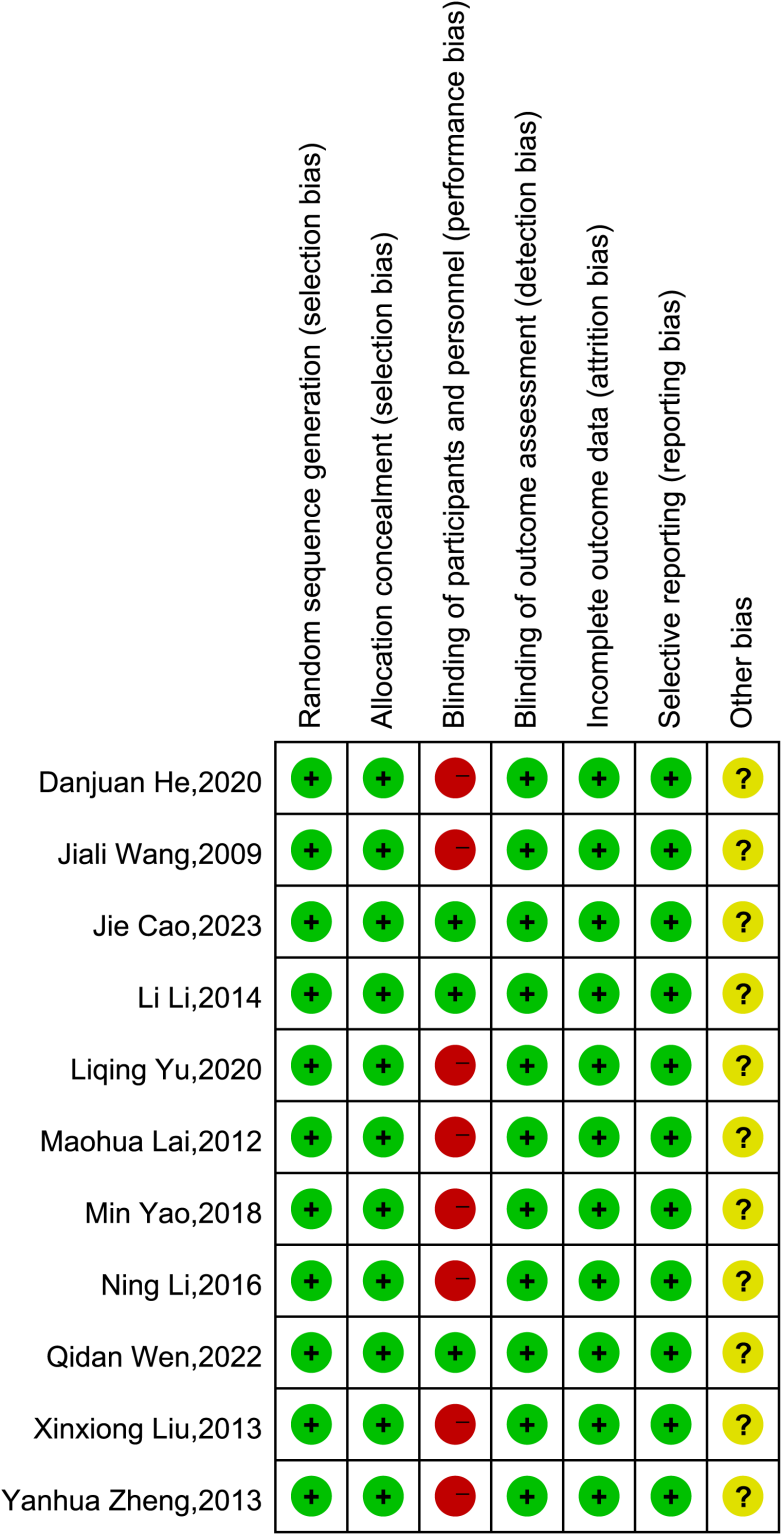
Assessment of risk biases of the included studies.

In four studies (22, 26, 28, 31), participants were randomly assigned using a random number table, while the remaining seven studies (21, 23–25, 27, 29, 30) only mentioned “randomization.” None of the studies reported allocation concealment. Given the nature of acupuncture and pharmaceutical treatments, blinding of participants was not feasible. Therefore, except for the three studies with placebo-controlled trials (29–31), which were deemed to have a low risk of bias, the remaining eight studies involving both participants and personnel blinding were classified as high risk (21–28). Outcome measurements were typically performed by a third party independent of the researchers, so blinding of outcome assessment was considered to be at low risk. Additionally, due to the lack of clarification in the articles, the risk of other biases for all 11 studies was classified as unclear. A detailed risk of bias assessment for each study is presented in **Figure 2**.

### 3.4 Meta-analysis results

#### 3.4.1 Meta-analysis of primary outcomes

**Figure 3:**
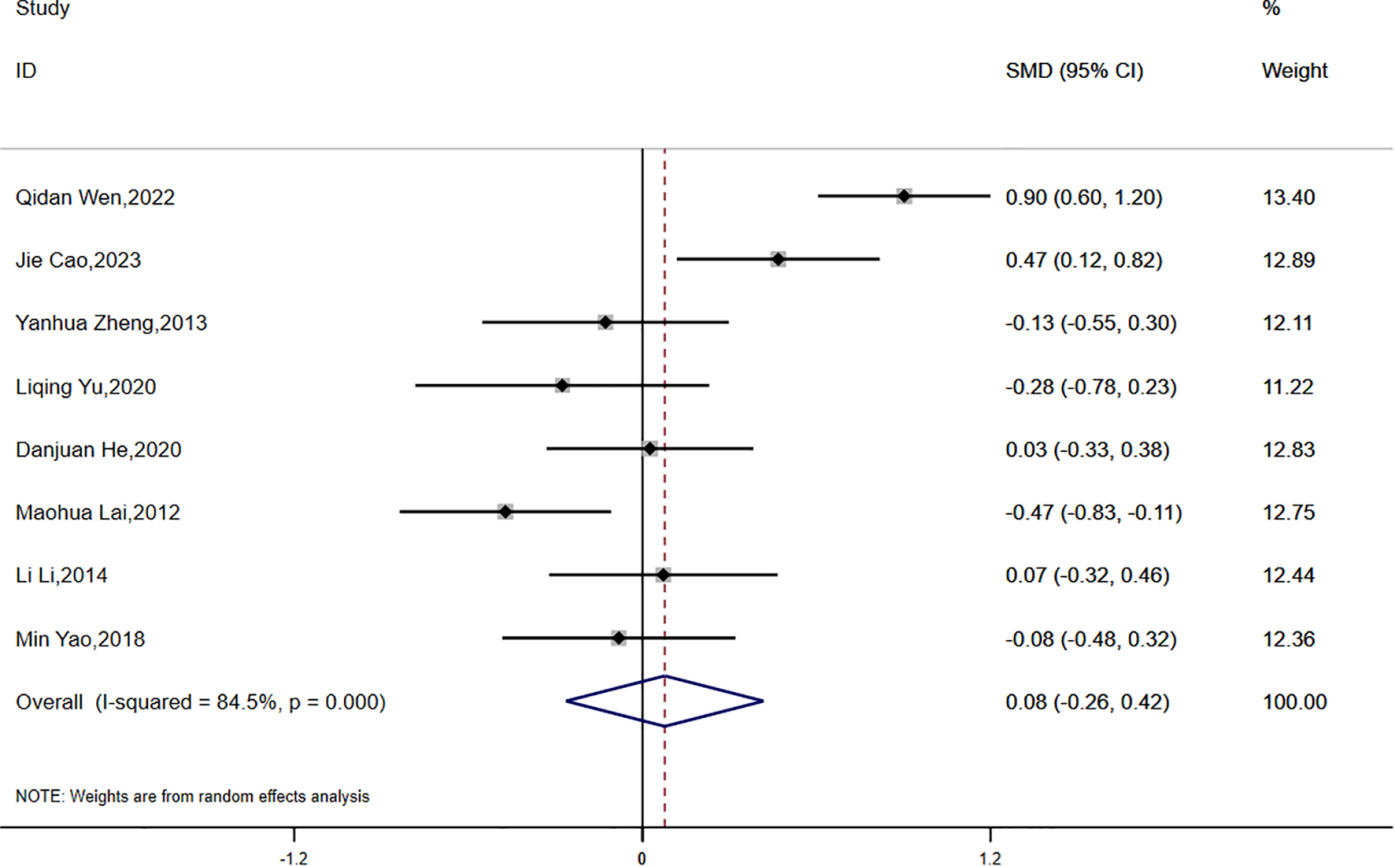
Comparison of changes in HOMA-IR according to intervention.

HOMA-IR:

Eight studies reported data on HOMA-IR, with significant statistical heterogeneity observed across the studies (P < 0.0001, I² = 84.5%). A random-effects model was applied for the meta-analysis. The results indicated that the reduction in HOMA-IR was smaller in the experimental group compared to the control group, although the difference was not statistically significant (SMD = 0.08, 95% CI [-0.26, 0.42]) (**Figure 3**).

#### 3.4.2 Meta-analysis of secondary outcomes

**Figure 4:**
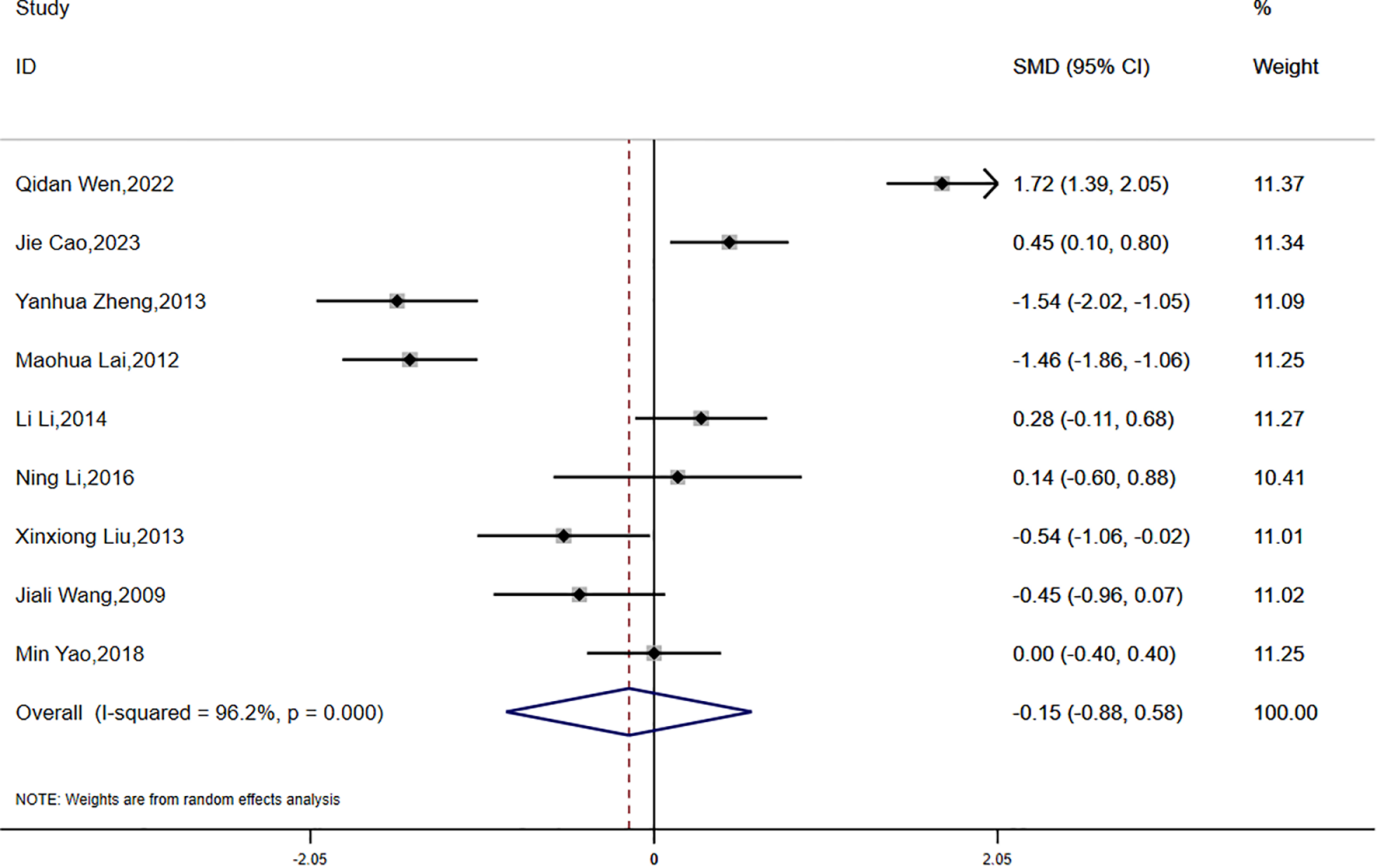
Comparison of changes in BMI according to intervention.

**Figure 5:**
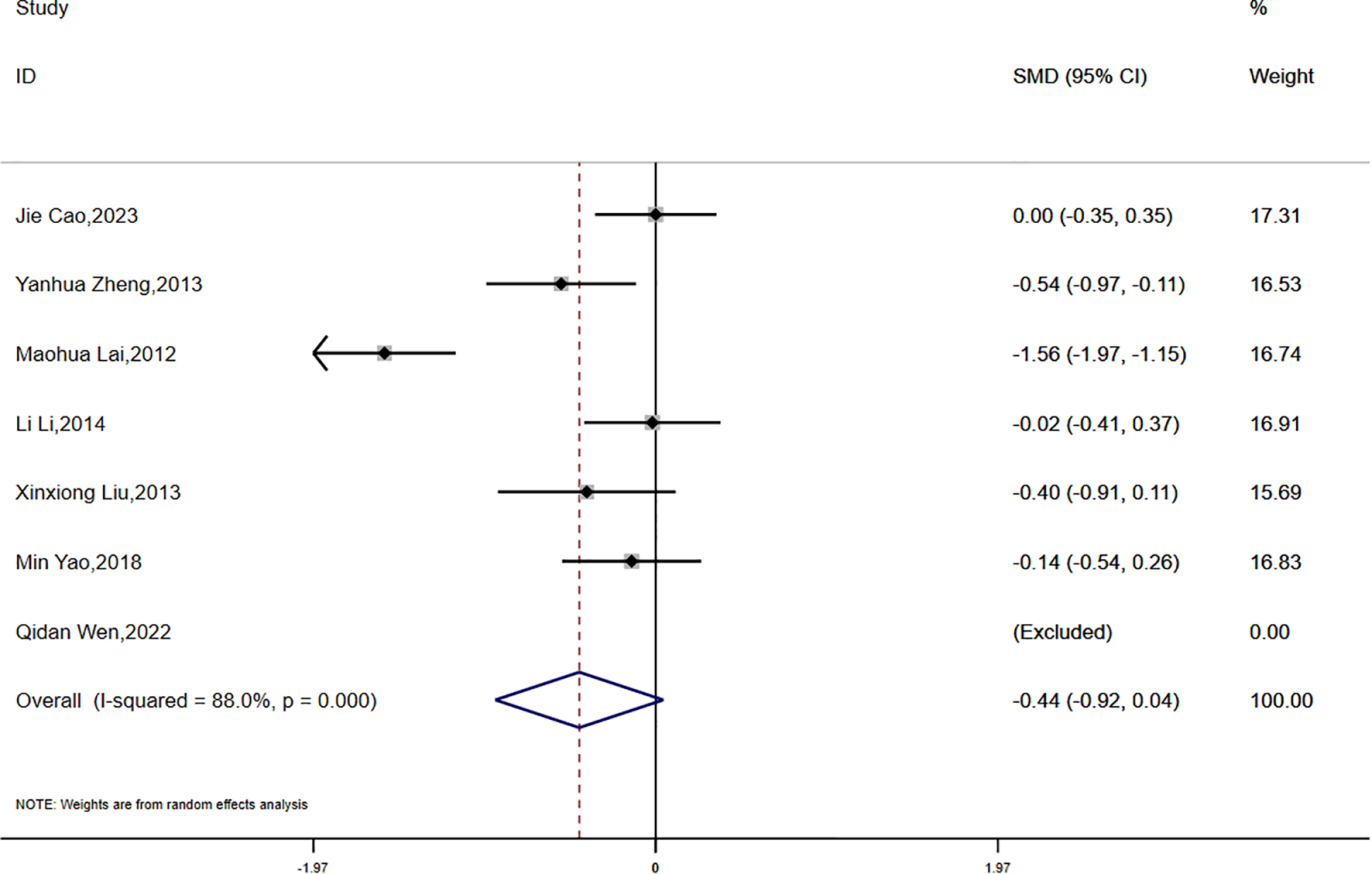
Comparison of changes in WHR according to intervention.

**Figure 6:**
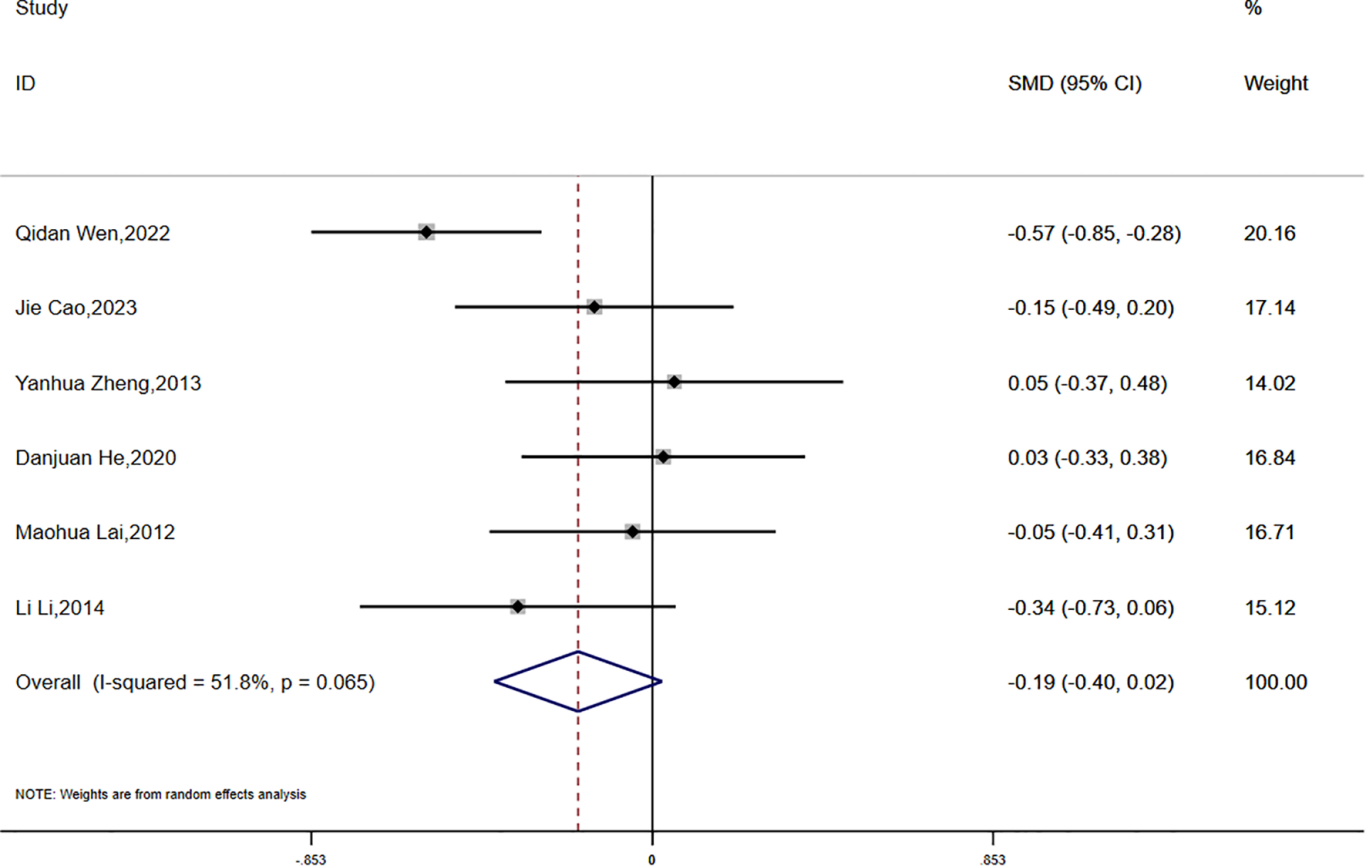
Comparison of changes in FPG according to intervention.

(1) BMI:

Data on BMI were reported in eight studies, which exhibited significant statistical heterogeneity (P < 0.0001, I² = 96.2%). Accordingly, a random-effects model was employed for the meta-analysis. The findings indicated a greater reduction in BMI in the experimental group compared to the control group; however, the difference was not statistically significant (SMD = -0.15, 95% CI [-0.88, 0.58]) (**Figure 4**).

(2)WHR:

Data on waist-to-hip ratio (WHR) were reported in seven studies, which demonstrated substantial statistical heterogeneity (P < 0.0001, I² = 88.0%). Consequently, a random-effects model was utilized for the meta-analysis. The analysis showed a greater reduction in WHR in the experimental group compared to the control group; however, the difference did not reach statistical significance (SMD = -0.44, 95% CI [-0.92, 0.04]) (**Figure 5**).

(3)FPG:

Fasting plasma glucose (FPG) was reported in six studies, which demonstrated moderate statistical heterogeneity (P = 0.065, I² = 51.8%). A random-effects model was applied for the meta-analysis. The results suggested a greater reduction in FPG in the experimental group compared to the control group; however, the difference did not reach statistical significance (SMD = -0.19, 95% CI [-0.40, 0.02]) (**Figure 6**).

(4)FINS

**Figure 7:**
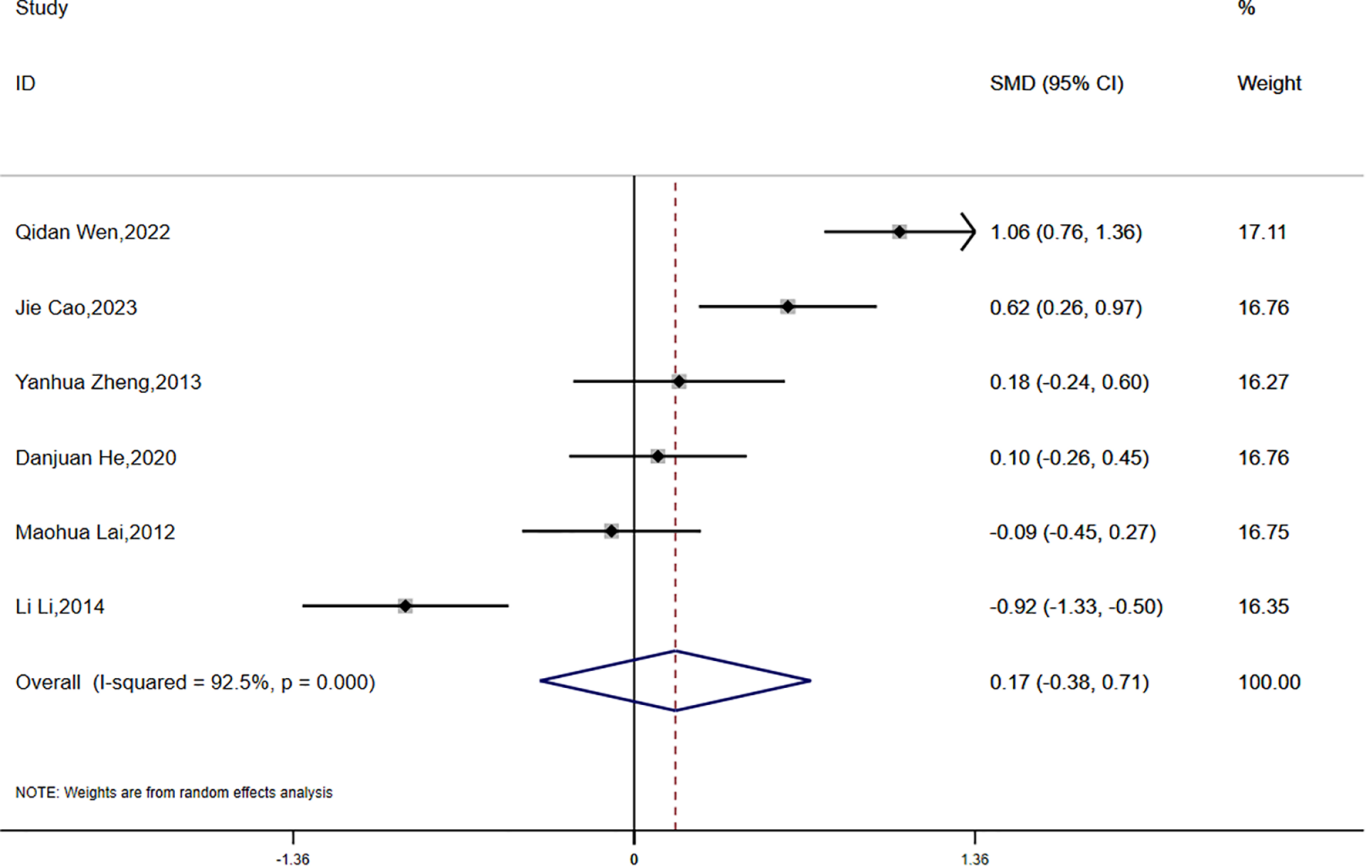
Comparison of changes in FINS according to intervention.

Fasting insulin (FINS) data were reported in six studies, revealing significant statistical heterogeneity (P < 0.0001, I² = 92.5%). A random-effects model was employed for the meta-analysis. The findings suggested that the reduction in FINS was smaller in the experimental group compared to the control group; however, this difference was not statistically significant (SMD = 0.17, 95% CI [-0.38, 0.71]) (**Figure 7**).

#### 3.4.3 Subgroup analysis

**Figure 8:**
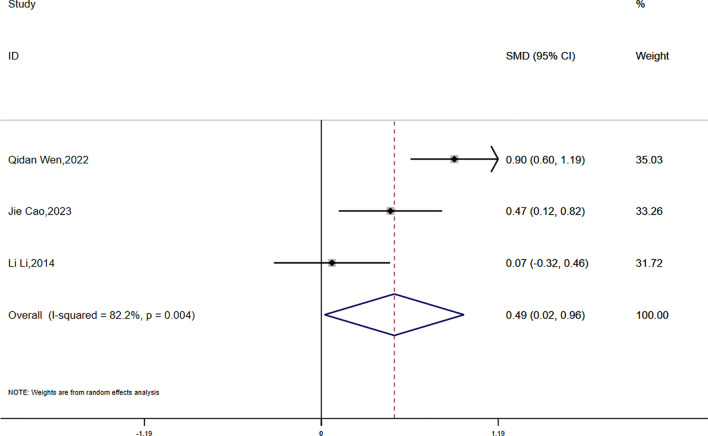
Comparison of changes in HOMA-IR according to intervention.

Meta-analysis of Primary Outcomes:

HOMA-IR:

Data on HOMA-IR were reported in three studies, which exhibited significant statistical heterogeneity (P = 0.004, I² = 82.2%). A random-effects model was used for the meta-analysis. The findings showed that the reduction in HOMA-IR was smaller in the experimental group compared to the control group, and this difference was statistically significant (SMD = 0.49, 95% CI [0.02, 0.96]) (**Figure 8**).

**Figure 9:**
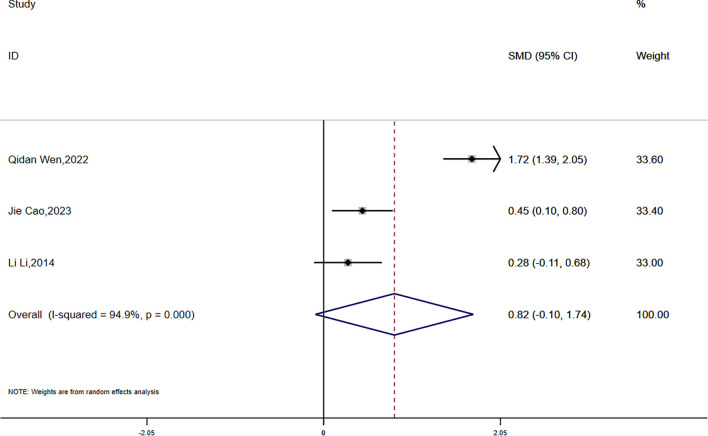
Comparison of changes in BMI according to intervention.

Meta-analysis of Secondary Outcomes:

(1) BMI:

Data on BMI were reported in three studies, which showed significant statistical heterogeneity (P = 0.000, I² = 94.9%). A random-effects model was employed for the meta-analysis. The findings suggested that the reduction in BMI was smaller in the experimental group compared to the control group; however, the difference was not statistically significant (SMD = 0.82, 95% CI [-0.10, 1.74]) (**Figure 9**).

**Figure 10:**
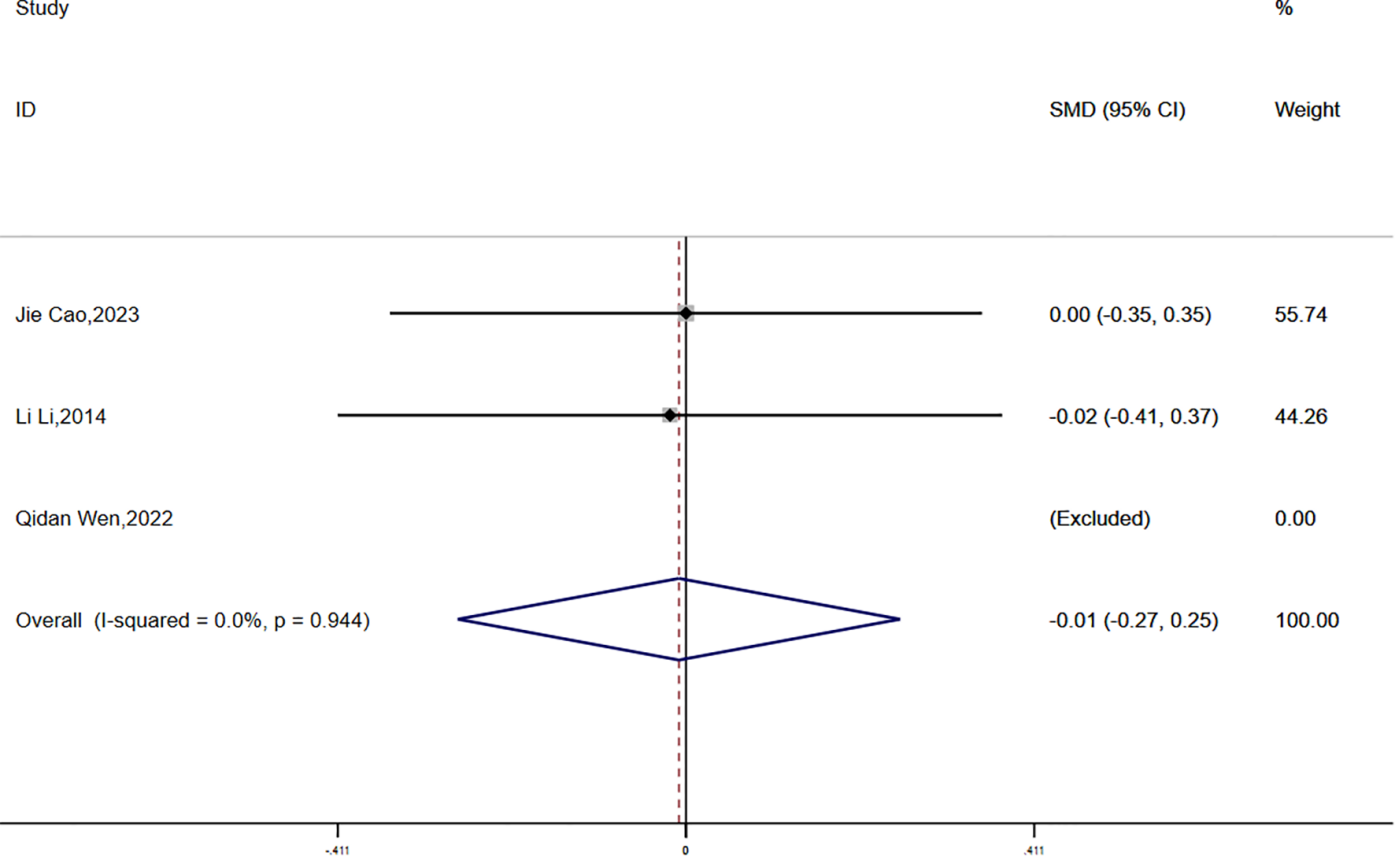
Comparison of changes in WHR according to intervention.

(2) WHR:

Waist-to-hip ratio (WHR) data were reported in three studies, with no statistical heterogeneity detected (P = 0.944, I² = 0.0%). A fixed-effects model was employed for the meta-analysis. The results demonstrated a greater reduction in WHR in the experimental group compared to the control group; however, the difference was not statistically significant (SMD = -0.01, 95% CI [-0.27, 0.25]) (**Figure 10**).

**Figure 11:**
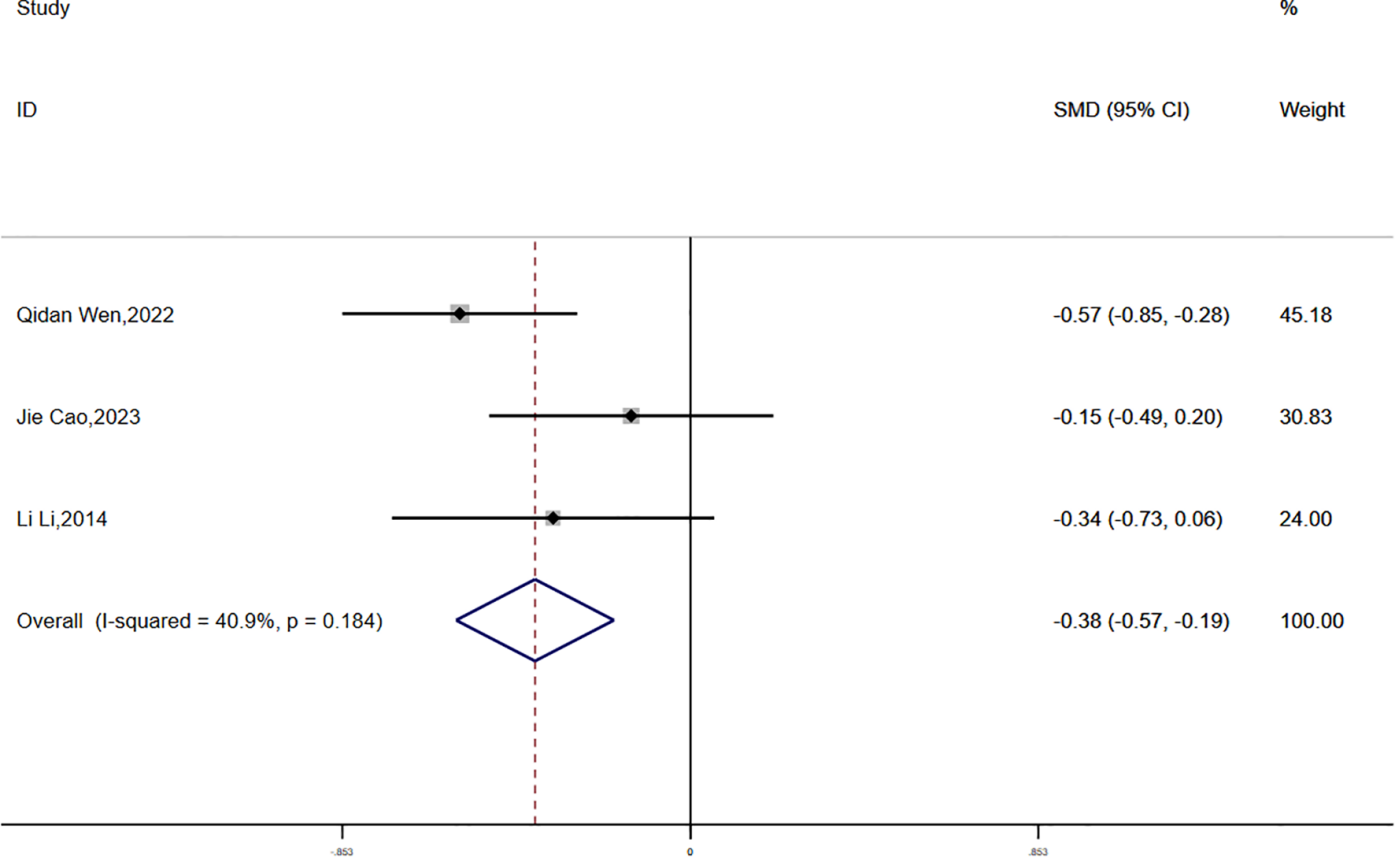
Comparison of changes in FPG according to intervention.

(3) FPG:

Fasting plasma glucose (FPG) data were reported in three studies, with no significant statistical heterogeneity detected (P = 0.184, I² = 40.9%). A fixed-effects model was applied for the meta-analysis. The findings demonstrated a greater reduction in FPG in the experimental group compared to the control group, and this difference was statistically significant (SMD = -0.38, 95% CI [-0.57, -0.19]) (**Figure 11**).

**Figure 12:**
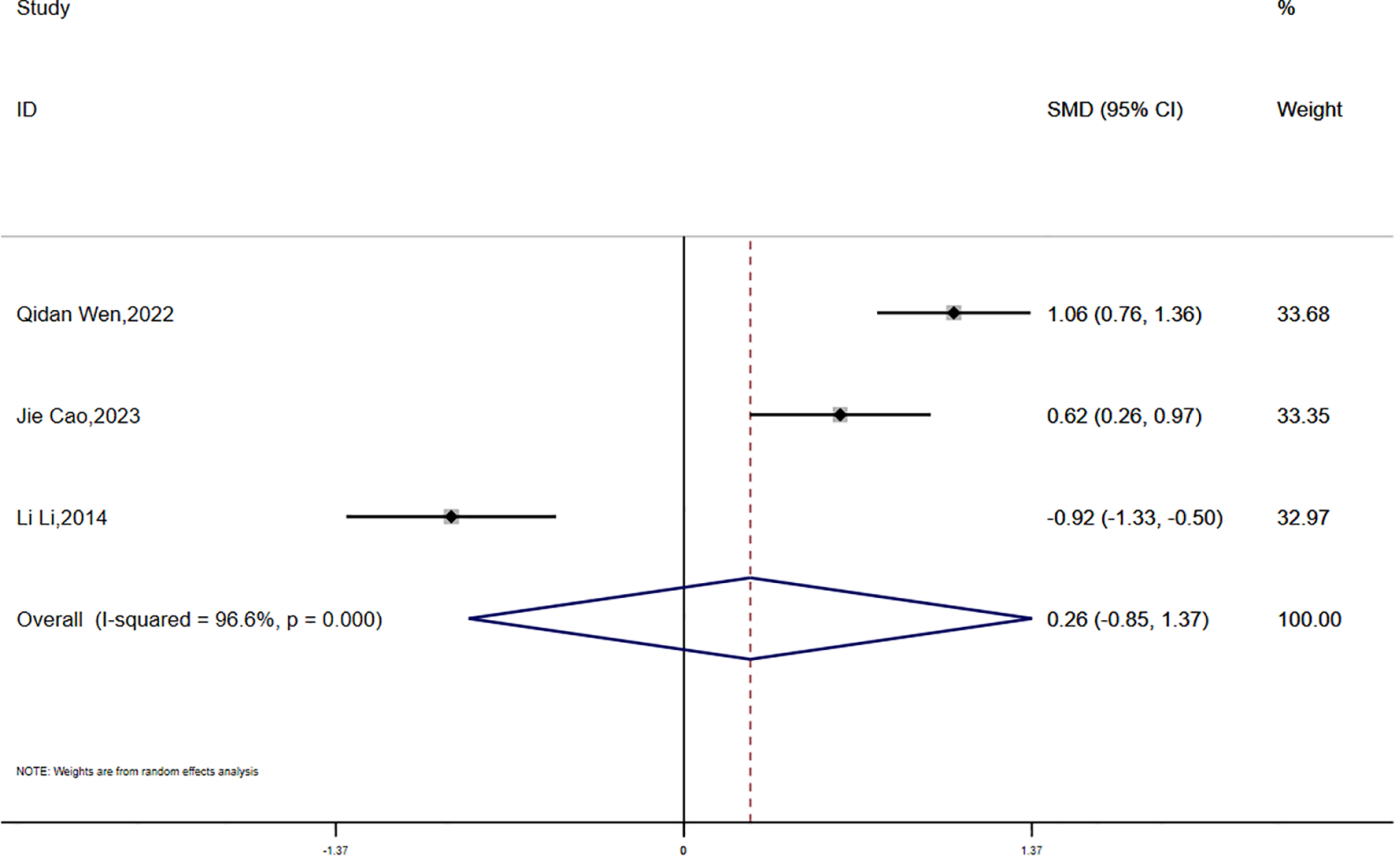
Comparison of changes in FINS according to intervention.

(4) FINS:

Fasting insulin (FINS) data were reported in three studies, showing significant statistical heterogeneity (P = 0.000, I² = 96.6%). A random-effects model was used for the meta-analysis. The results suggested that the reduction in FINS was smaller in the experimental group compared to the control group; however, the difference was not statistically significant (SMD = 0.26, 95% CI [-0.85, 1.37]) 7(**Figure 12**).

### 3.5 Network meta-analysis results

**Figure 13:**
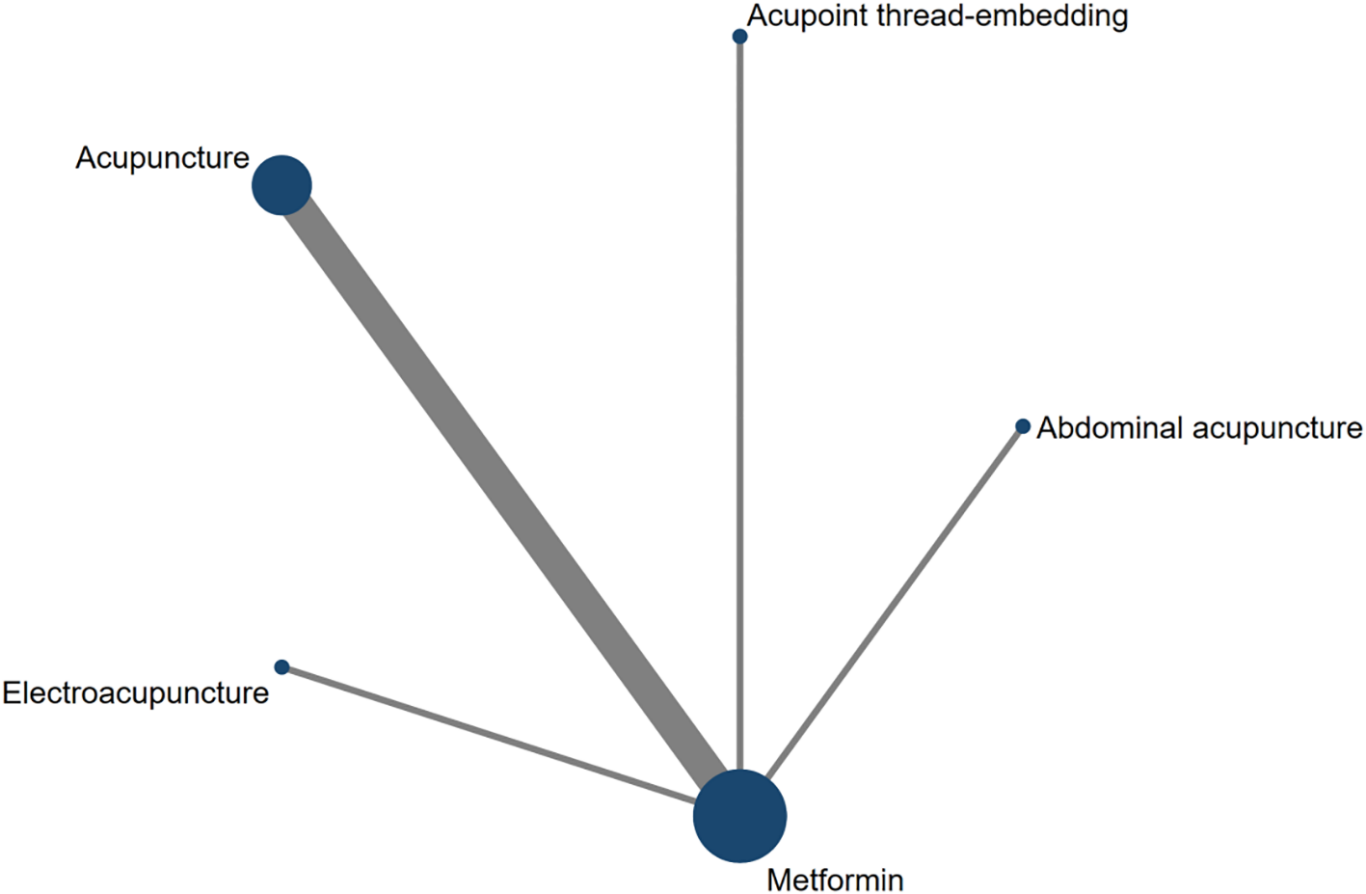
The network diagram of HOMA-IR.

Eight studies assessed HOMA-IR as an outcome measure. The network meta-analysis included five treatment modalities: Abdominal Acupuncture, Acupoint Thread-Embedding, Acupuncture, Electroacupuncture, and Metformin. The network structure of the interventions for the included subjects is depicted in **Figure 13**.

**Figure 14:**
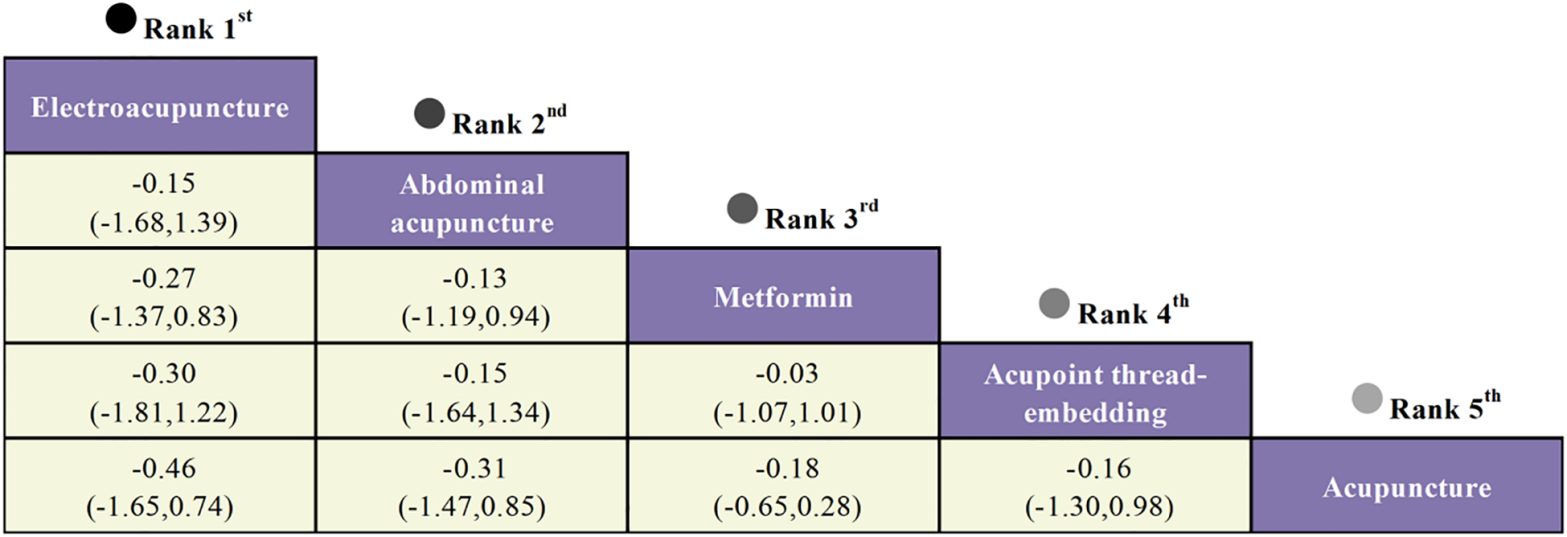
The league table of HOMA-IR.

The network meta-analysis indicated that no closed loop was established, necessitating the application of a consistency model for analysis. The analysis of standardized mean differences (SMD) and their corresponding 95% confidence intervals (CI) revealed that, compared to Metformin, only Electroacupuncture (SMD = -0.27, 95% CI [-1.37, 0.83]) and Abdominal Acupuncture (SMD = -0.13, 95% CI [-1.19, 0.94]) were associated with a significant reduction in HOMA-IR values in women with polycystic ovary syndrome. However, the inter-group differences were not statistically significant. Detailed results are provided in **Figure 14**.

**Figure 15:**
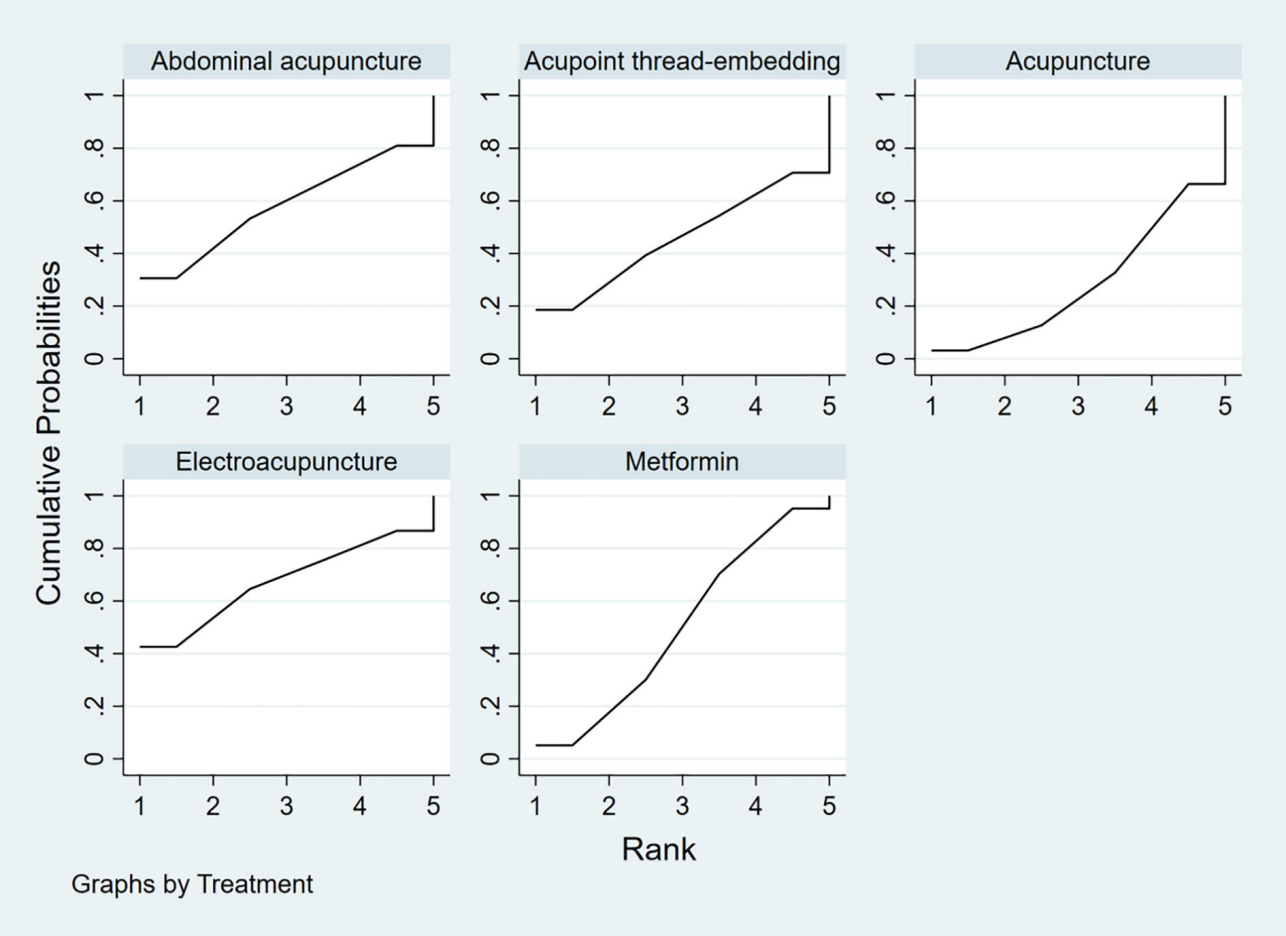
The rank chart of HOMA-IR.

To rank the interventions based on their efficacy in improving the HOMA-IR outcome, the interventions were ordered as follows: Electroacupuncture (SUCRA: 67.4%) > Abdominal Acupuncture (SUCRA: 58%) > Metformin (SUCRA: 50.2%) > Acupoint Thread-Embedding (SUCRA: 45.7%) > Acupuncture (SUCRA: 28.7%), as shown in **Figure 15**.

### 3.6 Meta-regressions

Meta-regression analysis, incorporating sample size, age, and follow-up duration as covariates, revealed that sample size was the only significant predictor of between-study heterogeneity (P = 0.032). In contrast, age (P = 0.368) and follow-up duration (P = 0.573) did not achieve statistical significance (α = 0.05).

### 3.7 Adverse events

Six studies (21, 22, 24, 28, 29, 31) reported the occurrence of adverse events, with all studies indicating gastrointestinal issues in the metformin group, such as nausea, vomiting, mild diarrhea, and slight dizziness. Three studies (22, 28, 29) also reported minor bruising at the acupuncture sites, with no other adverse events observed. **Table 2** displays the reported details.

**Table 2:** Incidence of adverse events included in the study.

studyID	Sample size (TG/CG)	Treatment	Control	Nausea, emesis, diarrhea, and loss of appetite		Subcutaneous bruising and hematoma	
				TG	CG	TG	CG
Wen, 2022 (29)	97/96	True acupuncture + Placebo	Metformin+sham acupuncture	—	100	17	—
Zheng, 2013 (21)	43/43	Abdominal acupuncture	Metformin	—	21	0	—
Yu, 2020 (22)	31/30	Electroacupuncture	Metformin	—	10	4	—
Lai,2012	60/60	Abdominal acupuncture	Metformin	—	12	0	—
Li, 2014 (31)	49/51	True acupuncture + Placebo	Metformin+sham acupuncture	—	22	0	—
Yao, 2018 (28)	48/48	Acupuncture	Metformin	—	11	1	—

TG, treatment group; CG, control group.

### 3.8 Sensitivity analysis

Sensitivity analysis was conducted using HOMA-IR, BMI, WHR, FPG, and FINS as indicators. After sequentially excluding individual studies, the results indicated that the heterogeneity did not significantly decrease compared to the initial analysis. This suggests that the findings of this study are robust.

## 4 Discussion

This study is the first to compare the effects of acupuncture and metformin monotherapy on insulin sensitivity in patients with polycystic ovary syndrome (PCOS). Based on assessments of HOMA-IR, BMI, WHR, FPG, and FINS, we found that metformin was more effective than acupuncture in reducing HOMA-IR, BMI, and FINS in PCOS patients, with a significant difference observed only in HOMA-IR. Acupuncture, on the other hand, showed superior effects in reducing WHR and FPG, with a significant difference observed only in FPG. The results of our network meta-analysis demonstrate that, compared to Metformin, only Electroacupuncture and Abdominal Acupuncture significantly reduced HOMA-IR values in women with polycystic ovary syndrome (PCOS), although the differences between groups were not statistically significant. Furthermore, when ranking the efficacy of different acupuncture modalities in improving HOMA-IR outcomes based on SUCRA scores, Electroacupuncture was identified as the most effective intervention for reducing HOMA-IR values in women with PCOS-related insulin resistance, followed by Abdominal Acupuncture. The adverse events reported with acupuncture (such as bleeding, bruising, etc.) were minor and transient, suggesting that acupuncture is a safe and reliable treatment.

Biguanide medications (such as metformin) are currently the most commonly used insulin sensitizers for treating insulin resistance in patients with PCOS, and they have been widely applied in improving glycemic control in these patients (32, 33). Acupuncture, as a non-pharmacological alternative treatment for PCOS, has shown beneficial effects on blood glucose outcomes, according to relevant meta-analyses (34), which is consistent with the findings of our study. A review by Zheng et al. reported that acupuncture can improve BMI, WHR, and HOMA-IR in PCOS patients, confirming its efficacy and safety in enhancing glucose metabolism and insulin sensitivity in this patient population (15).

In our study, when comparing acupuncture treatment with metformin therapy, we found that metformin was more effective in reducing HOMA-IR, while acupuncture demonstrated greater efficacy in lowering FPG. From a mechanistic perspective, the likely reason for this is that metformin, as an insulin sensitizer, enhances insulin sensitivity by inhibiting hepatic glucose production and suppressing gluconeogenesis and lipogenesis, which leads to a reduction in circulating insulin and glucose levels (35). Previous studies have shown that electroacupuncture improves insulin sensitivity by enhancing glucose tolerance and lowering fasting blood glucose levels in diabetic rats (36). Firouzjaei et al. confirmed that electroacupuncture combined with metformin treatment was more effective in reducing fasting blood glucose levels than metformin alone, suggesting that electroacupuncture may act similarly to an insulin sensitizer, potentially inducing hypoglycemic responses (17). Based on the results of this study, we conclude that acupuncture therapy is more advantageous in reducing FPG.

Furthermore, previous relevant meta-analyses on the effects of acupuncture on pregnancy rates and insulin resistance in PCOS patients often included studies involving combination therapies (16, 37). This study, however, only included clinical trials comparing standalone acupuncture treatment with metformin monotherapy, excluding studies on combined treatments. On one hand, by comparing the efficacy of acupuncture and metformin alone, this study provides evidence-based support for clinical practice. On the other hand, if acupuncture alone can achieve therapeutic outcomes without significant cost or time investment, additional treatments may not be necessary, and acupuncture could be considered a preferred non-pharmacological alternative therapy for PCOS patients. To compare the effects of different acupuncture methods on the HOMA-IR results, we ranked the intervention measures based on their SUCRA scores. Electroacupuncture was found to be the most effective intervention measure to reduce the HOMA-IR value in women with polycystic ovary syndrome (PCOS) -related insulin resistance, and this result is consistent with the meta-analysis result of Chen J et al (38). One possible explanation for this finding is that electroacupuncture may augment the effects of acupuncture points through electrical stimulation. Furthermore, the continuous nature of electroacupuncture stimulation is likely to be more consistent and sustained compared to manual needling, which may result in more substantial physiological responses. Previous research has indicated that electroacupuncture enhances systemic glucose uptake by stimulating both the sympathetic and parasympathetic nervous systems, a mechanism that could hold significant clinical implications for the management of insulin resistance (39).

Despite the important conclusions drawn from this study, several limitations should be noted. First, this study included only 11 randomized controlled trials (RCTs), most of which had small sample sizes. Second, acupuncture treatment could not be administered in a double-blind manner. Additionally, some of the included studies did not specify the randomization methods or failed to apply blinding procedures, which introduces certain limitations to the results. Finally, the studies included in this analysis exhibited variability in the diagnostic criteria for PCOS, leading to heterogeneity in population characteristics. Additionally, the interventions in the experimental groups, including acupuncture point selection and treatment frequency, were inconsistent across studies. Given the limited number of studies, it was not feasible to perform further subgroup analyses, which might have influenced the precision of the results.

While the advantages of metformin in improving insulin resistance in PCOS patients cannot be denied, acupuncture presents a viable alternative or adjunctive therapy outside of pharmacological treatments. However, there is limited information regarding the duration of acupuncture’s effects. Therefore, further research is needed to conduct long-term follow-up studies to evaluate the role of acupuncture in improving insulin resistance in PCOS, particularly its effectiveness in lowering FPG.

## Data Availability

The original contributions presented in the study are included in the article/**Supplementary Material**. Further inquiries can be directed to the corresponding author.
