# Supplementary material for: Comparative effects of acupuncture and metformin on insulin sensitivity in women with polycystic ovary syndrome: a systematic review and meta-analysis
Source: Front Endocrinol (Lausanne). 2025 Jun 18;16:1553684. doi: 10.3389/fendo.2025.1553684 (PMC12213467; doi:10.3389/fendo.2025.1553684)
Supplement: Supplementary file 1 [file DataSheet1.docx]

**Search Strategies**

**Pubmed：**

((("Polycystic Ovary Syndrome"[Mesh]) OR ((((((((((((((Ovary Syndrome, Polycystic) OR (Syndrome, Polycystic Ovary)) OR (Stein-Leventhal Syndrome)) OR (Stein Leventhal Syndrome)) OR (Syndrome, Stein-Leventhal)) OR (Sclerocystic Ovarian Degeneration)) OR (Ovarian Degeneration, Sclerocystic)) OR (Sclerocystic Ovary Syndrome)) OR (Polycystic Ovarian Syndrome)) OR (Ovarian Syndrome, Polycystic)) OR (Polycystic Ovary Syndrome 1)) OR (Sclerocystic Ovaries)) OR (Ovary, Sclerocystic)) OR (Sclerocystic Ovary)))

AND

(("Acupuncture"[Mesh]) OR ((((Pharmacopuncture) OR (electroacupuncture)) OR (manual acupuncture)) OR (catgut implantation at acupoint))))

AND

(("Metformin"[Mesh]) OR (((((((Dimethylbiguanidine) OR (Dimethylguanylguanidine)) OR (Glucophage)) OR (Metformin Hydrochloride)) OR (Hydrochloride, Metformin)) OR (Metformin HC)) OR (HCl, Metformin)))

**EMBASE:**

(exp polycystic ovary syndrome/ OR "polycystic ovary syndrome".ti,ab OR "polycystic ovarian syndrome".ti,ab OR "polycystic ovary disease".ti,ab OR "Stein-Leventhal Syndrome".ti,ab OR PCOS.ti,ab)

AND

(exp acupuncture/ OR exp moxibustion/ OR acupuncture.ti,ab OR electroacupuncture.ti,ab OR manual acupuncture.ti,ab OR "acupoint embedding".ti,ab)

AND

(exp metformin/ OR metformin.ti,ab)

AND

(exp insulin resistance/ OR "insulin resistance".ti,ab OR "insulin sensitivity".ti,ab)

AND

(exp randomized controlled trial/ OR "randomized controlled trial".ti,ab OR "randomised controlled trial".ti,ab OR randomied controlled trial.ti,ab OR RCT.ti,ab )

**Cochrane Library (CENTRAL):**

("Polycystic Ovary Syndrome"[MeSH] OR "polycystic ovary syndrome" OR "polycystic ovarian syndrome" OR "Stein-Leventhal Syndrome" OR PCOS)

AND

("Acupuncture Therapy"[MeSH] OR "Acupuncture" OR "Electroacupuncture" OR "Manual acupuncture" OR "acupoint embedding" )

AND

("Metformin"[MeSH] OR Metformin)

AND

("Insulin Resistance"[MeSH] OR "insulin resistance" OR "insulin sensitivity")

AND

("Randomized Controlled Trial" OR "Randomised Controlled Trial" OR RCT)

**Web of Science:**

TS = (("Polycystic Ovary Syndrome" OR "Polycystic Ovarian Syndrome" OR "Polycystic Ovary Disease" OR "Stein-Leventhal Syndrome" OR PCOS)

AND (acupuncture OR electroacupuncture OR manual acupuncture OR "acupoint embedding" )

AND (metformin )

AND ("insulin resistance" OR "insulin sensitivity" )

AND ("randomized controlled trial" OR "randomised controlled trial" OR RCT)

**CNKI/WanFang/VIP:**

(Polycystic Ovary Syndrome OR Polycystic Ovary Syndrome OR PCOS)

AND (Acupuncture OR electroacupuncture OR manual acupuncture OR acupoint acupuncture)

AND (Metformin)

AND (insulin resistance OR insulin sensitivity*)

AND (randomized controlled trial OR randomized controlled study OR RCT)
